# The History and Capabilities of Herbal Simples

**Published:** 1893-09-30

**Authors:** W. T. Fernie


### THE HISTORY AND CAPABILITIES OF HERBAL SIMPLES.

By W. T. Fernie, M.D.

*(Continued from page 326.)*

### THE GOOSEBERRY.

The gooseberry is botanically a magnified hairy currant, and is named "Grossularia" because resembling a small unripe fig, "grossulus." The French call it groseille, and the Scotch grosart. From being a prickly bush it has acquired the title with us of gorseberry, corrupted to gooseberry; or some persons derive this more remotely from kroosbar, a cross, because the triple spine of the fruit or berry presents the form of a cross. In our Eastern counties it grows wild about the hedges and thickets with a small poor berry, but is thought not to be a native fruit. There the cultivated fruit is called feapberry or thapeberry, and Norfolk schoolboys well know thape-pie, made from green gooseberries. In "Tabernæmontanus" the melon was figured to look like a large gooseberry, and was headed "Pfebe," and thus by a blunder this name became transferred to the gooseberry. Dietetically the fruit is admirably suitable for persons of plethoric build, of sedentary habits, and of a bilious disposition, especially gooseberries of the red and hairy sort, though the yellow variety has the richest and most vinous flavour. Chemically gooseberries contain malic and citric acids, with lime and with pectine, which converts their juice when boiled into jelly. As far as we know, our original gooseberry, the "uva crispa" of Fuschius—*tempore* Henry VIII.—was red and hairy like Esau. Gooseberries make an excellent vinegar, and the juice of the green fruit, when combined with spices, is used as a corrective sauce with mackerel and other rich fish. The light summer dish so popular with us, gooseberry fool, is made by admixing cream and milk with the fruit, "foulé," or beaten and crushed. Unripe gooseberries are said to abate the strange longings which sometimes beset parturient ladies. To "play old gooseberry" with any one is to take great liberties with his property, in fact, to make gooseberry fool of the same. Gerarde says the juice of green gooseberries cureth all inflammations, and the young tender leaves, if eaten in a salad, drive forth the gravel. Gooseberry wine, when skilfully made, has the colour and flavour of choice champagne. Said Goldsmith's dear old Vicar of Wakefield in his classic story, "Sometimes Farmer Flamborough, our talkative neighbour, and often the blind piper, would pay us a visit and taste our gooseberry wine, for which we had lost neither the recipe nor the reputation." In Lancashire gooseberry shows are held annually, and excite keen competition among the exhibitors. The prizes are awarded for the biggest and heaviest berries. For producing these, immense pains are taken to manure the shrubs properly, and a growth of cooling chickweed is encouraged round their roots, whilst each berry is kept constantly in a small bath of cold water so as to be always absorbing moisture. Southey, in "The Doctor," quotes from an old Manchester newspaper an obituary notice of a man who "bore a severe illness with Christian fortitude, and was highly esteemed among gooseberry growers." The "Gooseberry Book" is a regular Manchester annual, always much in demand. A berry has been produced there which turned thirty-seven pennyweights in the scale; and a tale is recorded of a certain Middleton weaver who, when a thunderstorm seemed to threaten at night, lay awake as if for his life, and at the first patter of rain against the window-panes rushed to the rescue of his bushes with his bed quilt. Likewise in Scotland the fruit is carefully cultivated, but it grows better in Great Britain than anywhere else because of our cool humid climate. And undoubtedly the best method of producing this fruit is by training the shrubs upon trellises. Some writers say, thoughtlessly, that gooseberries are so named because eaten as sauce with young or green geese. Others attribute to the Norfolk term "feapberry" a meaning of feverberry, from the juice being beneficial in fever. The French "sirop de groseille," so eagerly asked for on the boulevards, is made, after all, from the juice of red currants. "You were a goose, Berry," said comic Bob Keeley to Sam Berry at the Adelphi in my salad days, "to make such a mull, Berry, as to sign that bill, Berry, before it was due, Berry," and so on, with a long, clever play on divers berries and his friend's most convenient name. Alas, poor Yorick! Where be your merry jests now? In Devon the rustics call gooseberries "deberries," whilst to lads in Sussex they are familiarly known as goosegogs. An Irish cure for warts is to prick them with a gooseberry thorn passed through a wedding-ring. The poet Southey wrote "A Pindaric Ode upon a Gooseberry Pie":—

"And didst thou scratch thy tender arms,
O gatherer?" &c., &c.

### AN HYGIENIC MASK.

The Industrial Association of France have done good service in proposing for public competition the invention of a good type of respirator mask to serve as a protection to the workman against imbibing injurious particles of dust. This respirator mask must fulfil the following conditions: (1) It must efficaciously protect the mouth and nose of the workman from the absorption of dust particles. (2) Without being fragile, it must be lightly and easily put on. (3) It must be inexpensive, and easily cleaned. (4) It must neither impede the breathing nor heat the face. A special commission will sit to decide upon the award.

